# Aptamer-Based Strategies to Address Challenges in COVID-19 Diagnosis and Treatments

**DOI:** 10.1155/2023/9224815

**Published:** 2023-07-31

**Authors:** Zi Yuan Chang, Falah Abbas Mohamad Salih Alhamami, Kai Ling Chin

**Affiliations:** ^1^Department of Biomedical Sciences, Faculty of Medicine and Health Sciences, Universiti Malaysia Sabah, Kota Kinabalu, Sabah, Malaysia; ^2^College of Medicine, AL-Warith University, Karbala, Iraq

## Abstract

Coronavirus disease (COVID-19), a highly contagious and rapidly spreading disease with significant fatality in the elderly population, has swept across the world since 2019. Since its first appearance, the causative agent, severe acute respiratory syndrome coronavirus 2 (SARS-CoV-2), has undergone multiple mutations, with Omicron as the predominant circulating variant of concern at the moment. The gold standard for diagnosis of COVID-19 by real-time polymerase chain reaction (RT-PCR) to detect the virus is laborious and requires well-trained personnel to perform sophisticated procedures. Also, the genetic variants of SARS-CoV-2 that arise regularly could result in false-negative detection. Meanwhile, the current COVID-19 treatments such as conventional medicine, complementary and alternative medicine, passive antibody therapy, and respiratory therapy are associated with adverse effects. Thus, there is an urgent need to discover novel diagnostic and therapeutic approaches against SARS-CoV-2 and its variants. Over the past 30 years, nucleic acid-based aptamers have gained increasing attention and serve as a promising alternative to the antibodies in the diagnostic and therapeutic fields with their uniqueness of being small, nonimmunogenicity, and thermally stable. Aptamer targeting the SARS-CoV-2 structural proteins or the host receptor proteins represent a powerful tool to control COVID-19 infection. In this review, challenges faced by currently available diagnostic and therapeutic tools for COVID-19 are underscored, along with how aptamers can shed a light on the current COVID-19 pandemic, focusing on the critical factors affecting the discovery of high-affinity aptamers and their potential applications to control COVID-19 infection.

## 1. Introduction

Coronavirus disease 2019 (COVID-19) is caused by a novel beta coronavirus (*β*-CoV) called severe acute respiratory syndrome coronavirus 2 (SARS-CoV-2). The sudden outbreak of the disease in 2019 has tremendously impacted the global economy and human well-being. On 11 March 2020, the COVID-19 infection is declared a pandemic by the World Health Organization (WHO). To date, more than 676 million cases and nearly 7 million deaths were reported [1]. Since the emergence of the COVID-19 pandemic, the scientific endeavor has focused on developing diagnostic tools and novel therapies to control the disease. One of the strategies being pursued is through the discoveries of novel ligands that either target the structural proteins of the SARS-CoV-2 [2–9] or the host proteins [10].

Antibody is one of the promising binding agents that has been frequently used in diagnostic and therapeuti [11]. Hybridoma technology is one of the most common methods used to produce monoclonal antibodies (mAbs) by utilizing *in vivo* immunization with an antigen to provoke an immune response, followed by isolation and fusion of B-cells with myeloma cells to produce hybridomas, and subsequently screening for antigen-specific mAb-producing immortal hybridoma clones [12]. Alternatively, high-affinity reagents which are nonanimal technologies, i.e., recombinant antibodies and aptamers are widely used to replace the animal-based mAbs [13]. Recombinant mAbs are protein-based reagents produced by a screening method known as phage display [14], while aptamers are nucleic acid-based binders selected using the systematic evolution of ligands by exponential enrichment (SELEX) method [15, 16]. Both approaches are robust and inexpensive techniques to discover specific antigen binders from a large combinatorial library. The high flexibility of the combinatorial libraries allows the screening of potential recognition agents to be performed without prior knowledge about the characterization of the target structure.

SELEX differs from the phage display library technique as the former is a combinatorial chemistry approach, while the latter is a combinatorial biology approach. The phage display has a unique advantage allowing complete characterization of the antibody in just 2 to 4 cycles of selection. However, its application has been largely constrained by its small library diversity (∼10^11^), attributed to several practical factors that can limit the effective size of the library [14]. In contrast, the combinatorial library of SELEX generally can contain up to 10^15^ unique aptamers. The aptamer not only can bind to its target with a binding affinity that is comparable to an antibody, but also poses many superior properties over mAb including smaller size, thermal stability, nonimmunogenetic, and ability to regenerate without batch-to-batch variation [17].

Thus, considering all the advantages of the aptamers over the antibodies, SELEX has been used to identify structured aptamers for the recognition of targets to be employed as detecting and therapeutic agents. However, not many aptamer-based products are commercially available due to the industrial commitment to antibodies and ignorance of aptamer capabilities [18]. Nevertheless, selecting sophisticated aptamers is not easy as their development is highly dependent on several critical conditions during the *in vitro* SELEX process. Here, we will review the critical factors related to the successful isolation/selection of the aptamers and their application in the diagnosis and treatment of COVID-19.

## 2. Pathogenesis of COVID-19

Angiotensin-converting enzyme 2 (ACE2) protein is predominately found at the apical surface of cells to act as a type I transmembrane protein [19, 20]. This transmembrane protein comprises an ectodomain region that contains an active site that can interact with spike proteins (S proteins) of coronaviruses with high affinity. During the engagement process to ACE2, the SARS-CoV-2 move in hinge-like conformational rhythms such that during its “up” conformation, the virus exposes its receptor-binding domains (RBDs) on the S protein for receptor engagement, while hiding its RBDs during “down” conformation to transiently evade host immunological recognition [21, 22]. Attachment of the SARS-CoV-2 S protein to ACE2 triggers host transmembrane protease serine 2 (TMPRSS2) to prime the cleavage of the S protein into S1 and S2 domains. This priming process activates the S2 domain to initialize the fusion process of the virus into the cell to undergo replication [23]. Also, a polybasic furin cleavage site which is located in between the S1 and S2 subunits promotes the fusion of the virus particles into the human lung cells by facilitating in the conformation change of the S proteins [21].

SARS-CoV-2 fuses with the host plasma membrane and releases the viral RNA into the cell. The viral genome undergoes replication by hijacking the host machinery. The viral genome is then translated to proteins where the first two-thirds of the viral genome encode 16 nonstructural proteins. These nonstructural proteins are needed for the replication of subgenomic messenger RNAs that encode nine accessory proteins open reading frames (ORFs) (3a, 3d, 6, 7a, 7b, 8, 9b, 14, and 10) and four structural proteins (S protein, membrane protein (M protein), envelope protein (E protein), and nucleocapsid protein (N protein)) [24].

RNA interference (RNAi) pathway is an antiviral immune defense mechanism of the host cell to destruct the viral genome [25]. The N protein acts as a viral suppressor of RNAi, allowing the viral genome to replicate effectively [26]. Also, the N protein interacts with the M protein to facilitate virus assembly. The M protein as well as the E protein and S protein have trafficking signal sequences that translocate them to the endoplasmic reticulum (ER) [27]. These structural proteins together with the viral genome are then assembled into virus particles in the host ER-Golgi intermediate compartment. The vesicle-containing virus then fuses with the host's plasma membrane and is released from the cell by exocytosis to infect more host cells.

Infection by the SARS-CoV-2 will downregulate the ACE2 expression at the cell surface as the ACE2 will internalize together with the virus into the cell through endocytosis [28]. Since ACE2 plays as a negative regulator in the renin-angiotensin system, by converting the waste products of ACE (i.e., Angiotensin I and Angiotensin II) into inactive peptides [20, 29], loss of ACE2 catalytic functions due to the SARS-CoV-2 can cause a series of damages on the lungs [30]. These damages include increased vascular permeability to enhance the viral entry and promote SARS-CoV-2-mediated acute respiratory distress syndrome (ARDS) in the lungs [28, 31].

ARDS can also be driven by cytokine storm [32]. Cytokine storm happens when immersed amounts of cytokines are released into the system due to aberrant host immune response towards high loads of SARS-CoV-2 in the body. Generally, virus replication will activate innate immune responses when the viral RNA is detected by endosomal RNA receptors (i.e., toll-like receptors) or cytosolic RNA sensors (i.e., retinoic acid-inducible gene I and melanoma differentiation associated protein 5). However, SARS-CoV-2 poses several unique features to evade host immunological defense. For example, the virus accessory protein ORF-9b can bind to the host mitochondrial antiviral-signaling protein (MVSP), a protein needed to trigger the secretion of interferons to interfere with the replication of SARS-CoV-2 [33]. On the other hand, it has been reported that two accessory proteins of SARS-CoV-2 (ORF6 and ORF8) and the N protein are potent interferon antagonists [34]. Delayed signaling or reduced expression of these interferons allows the virus to replicate uncontrollably in the cells. Once the infected cells undergo cell death, high loads of virus particles will be released, resulting in hyperinflammatory immune responses as aberrant amounts of proinflammatory cytokines such as interleukin-6 (IL-6) and tumor necrosis factor-alpha (TNF-*α*) are secreted for the recruitment of more immune cells to the site of infection [33, 35]. The ARDS reduces oxygen supply from the alveoli to the blood, leading to hypoxemia which eventually can cause multiple organ failure [36].

SARS-CoV-2 primarily causes lung infection. Nonetheless, SARS-CoV-2 can also lead to systemic infection in several major organs. This viral dissemination of SARS-CoV-2 to other organs is evidenced by the fact that the COVID-19 patients also developed nonrespiratory-related manifestations [37]. Notably, most of the COVID-19 patients frequently experience neurological-related symptoms such as brain fog and olfactory dysfunction, while a minority of the patients present gastrointestinal tract-related symptoms such as nausea, heartburn, and diarrhea. The widespread expression of the ACE2 receptor on a variety of cell types, including endothelial, neuronal, and epithelial cells, suggested the invasion route of SARS-CoV-2 to the central nervous system [37, 38]. SARS-CoV-2 invades the brain either via the olfactory route by infecting the olfactory neuron or through cerebral circulation to infect meningeal endothelial lining before invading the brain. The unprecedentedly high incidence of neurological alternation observed in COVID-19 patients is believed due to the uniqueness of furin cleavage site owned by the SARS-CoV-2 S protein. The furin cleavage site gives a slightly positive net charge to the S protein which can promote their crossing over the blood-brain barrier, thus enhancing the neurotropism of the SARS-CoV-2 in the brain [38]. Invasion of the SARS-CoV-2 to the brain causes local inflammation induced by the elevated level of proinflammatory cytokines, resulting in reversible mild symptoms (i.e., olfactory dysfunction) or severe longer-lasting brain damage which can trigger neurological syndromes (i.e., stroke and Parkinson's disease) [39, 40].

## 3. Challenges in the Diagnosis and Treatment of COVID-19

Previously, the *β*-CoVs have been known as inconsequential pathogens and received relatively little attention as the virus mainly induces flu with mild symptoms in healthy humans. However, this situation had changed in the 21st century with the emergence of several highly pathogenic *β*-CoVs originating from animal reservoirs [41]. The emergence of *β*-CoVs, namely, severe acute respiratory syndrome coronavirus (SARS-CoV), Middle East respiratory syndrome coronavirus (MERS-CoV), and SARS-CoV-2 have caused worldwide concern on the development of zoonotic diseases, i.e., severe acute respiratory syndrome (SARS), Middle East respiratory syndrome (MERS), and COVID-19, respectively. SARS was first detected in February 2003 and there were no reported SARS cases since 2004 [42]. MERS was first identified in September 2012 and this disease is still ongoing, but its outbreak remains epidemic due to its limited transmission rate among humans [43]. COVID-19 was first detected in December 2019, and it continues to spread globally at an unprecedented rate as the virus keeps evolving rapidly. On 26th November 2021, WHO declared the newly emerged Omicron (B.1.1.529) as the fifth SARS-CoV-2 variant of concern (VOC). This variant is the most mutated SARS-CoV-2 variant and has spread more rapidly than the previous VOC: Alpha (B.1.1.7), Beta (B.1.351), Gamma (P.1), and Delta (B.1.617.2) [44]. Omicron has a higher basic reproduction number (*R*_0_) than the Delta variant with an *R*_0_ = 5.08 [45]. *R*_0_ represents the average number of new infections caused by an infected individual, and *R*_0_ > 1 implies that the transmission is likely to increase. This pandemic situation undoubtedly makes the containment of COVID-19 a constant challenge, especially in the diagnosis and treatment of COVID-19.

## 4. Diagnosis of COVID-19

The diagnosis of COVID-19 includes the detection of host antibodies to the viral antigens and the detection of viral nucleic acids and antigens. During the early outbreak of COVID-19, serological and nucleic acid amplification tests (NAATs) are the two main laboratory diagnostic tests for the disease [46]. The serological tests detect the presence of IgM and IgG antibodies in human sera against the viral S protein or N protein that have high sensitivity to diagnose subclinical and late infections, respectively [47]. However, serological tests have low specificity due to cross-reactivity with other human coronaviruses. In 2021, De Assis et al. developed a high-throughput screening assay named coronavirus antigen microarray (COVAM) for COVID-19 serologic surveillance [48]. COVAM contains nearly 70 immunologically significant antigens from both the S protein and N protein of seasonal human coronaviruses and SARS-CoV-2 to detect antibodies against these coronaviruses simultaneously. This microarray demonstrated low cross-reactivity and the ability to discriminate COVID-19 antibody profiles. Nonetheless, serological tests are not suitable for early COVID-19 detection as the antibodies against the SARS-CoV-2 typically developed after one week of postinfection. Also, the antibodies are detected during the disease course and after the symptomatic phase, resulting in uncertainty on the utility of the tests for seroprevalence surveys for public health management purposes [49].

Advancement in molecular diagnostics has enabled the development of a rapid and portable diagnostic test called the rapid test kit antigen (RTK-Ag). This rapid lateral flow immunoassay-based assay serves as an alternative to the NAATs by quantitatively detecting the presence of the viral N proteins. Its accepted sensitivity to detect symptomatic cases within 15 minutes has certainly increased total per capita testing rates and alleviated NAATs workloads by providing point-of-care testing [50, 51]. Besides, RTK-Ag is expected to resist the SARS-CoV-2 synonymous mutation. Nevertheless, RTK-Ag has low sensitivity to pick up the asymptomatic cases, as its sensitivity is dependent on the viral load [52]. Hence, the NAATs remain the pillar of COVID-19 diagnostics, while the RTK-Ag is considered a preliminary screening test.

The NAATs which detect the viral RNA are the only diagnostic method for early onset of COVID-19 infection. Real-time polymerase chain reaction (RT-PCR) was first exploited for COVID-19 detection [53]. The RT-PCR has the merit of providing extremely sensitive, specific, and quantitative detection of COVID-19 at the same time allowing high-throughput testing of samples. Nonetheless, this approach is expensive, time-consuming, and require well-trained clinical laboratory personnel [54]. Hence, several isothermal amplification methods such as reverse transcriptase loop-mediated isothermal amplification, rolling circle amplification, multiple cross displacement amplification, nucleic acid sequence-based amplification, and recombinase polymerase amplification have been incorporated into COVID-19 detection to conquer the limitations of RT-PCR. These isothermal amplification methods can efficiently amplify nucleic acid at a constant temperature without the requirement of sophisticated equipment. These methods have been coupled to portable optical and electrochemical biosensors to provide highly sensitive diagnostic tests for COVID-19 [55].

The sensitivity of NAATs varies by test, but the laboratory-based RT-PCR generally has higher analytical sensitivity than those in-field tests [56, 57]. Despite the RT-PCR represents the gold standard for the diagnosis of COVID-19, its diagnostic accuracy could be impacted by the quantity and quality of viral RNA, resulting in false-negative detection [58, 59]. The high erroneous results of COVID-19 can occur due to numerous reasons including suboptimal sample collection [60], inappropriate sample collection time [61], low viral load [62], and handling error during the processing of the biological sample that could result in the degradation of the RNA [63].

Apart from that, the current COVID-19 diagnosis is also challenged by the mutating SARS-CoV-2. The current main circulating VOC, Omicron, has the highest transmission rate. It is the most mutated SARS-CoV-2 variant with several of its descendent lineages having been classified by WHO as variants of interest (XBB.1.5 and XBB.1.16) and variants under monitoring (BA.2.75, CH.1.1, BQ.1, XBB, XBB.1.9.1, XBB.1.9.2, and XBB.2.3) [44]. Omicron has over 50 mutations reported, mainly spotted in the spike and nucleocapsid genes [64]. These two genes are the main target analysts of RT-PCR [65]. Mutations in these genes have caused the failure of detection due to a mismatch between the target and primer sequences (Food and Drug Administration [66]). This mismatch can reduce the thermal stability of the primer-sample duplex and reduce the polymerization of the sample during the PCR reaction, resulting in decreased sensitivity in detecting new mutating Omicron variants [67].

The development of next-generation sequencing (NGS) and bioinformatics have enabled millions of viral samples to be sequenced independently and simultaneously in a short time and at a lower cost for early detection and accurate identification of SARS-CoV-2 variants at an unprecedented rate. The application of NGS enables routine genomic surveillance of the virus to monitor their evolution which may compromise the effectiveness of existing detection methods [68]. The high transmissibility pattern of the circulating Omicron sublineages undoubtedly presents a colossal new challenge in COVID-19 detection as there is always a portentous need for the manufacturer to regularly optimize the diagnostic primers to ensure the sensitivity and accuracy of the diagnosis method.

## 5. Treatment of COVID-19

The clinical manifestations of COVID-19 infection range from asymptomatic to critical illness. Asymptomatic or mild cases can be self-managed by getting adequate rest and nutrition along with practicing social distancing, while severe cases require immediate medical intervention. Hospitalized COVID-19 patients have primarily been managed empirically, focusing on providing supportive care and repurposing existing drugs [69]. The purpose of drug repurposing is to discover new clinical use for existing medications for COVID-19 infection that have already been approved as safe for people. This approach acts as a cost-effective, quick response to the COVID-19 infection as it does not need to be tested in the preclinical phases I and II and could be used directly in clinical trials [70].

SARS-CoV-2 replication is responsible for early pathogenesis, while the abnormal host immune response drives the late clinical course of the COVID-19 infection [71]. Hence, depending on the phases of COVID-19 infection, different therapeutic classes are used for the treatment of the disease [72] (Table 1). For example, antiviral medication (i.e., remdesivir) that interrupts the viral replication pathway has shown effectiveness in the early treatment of COVID-19 infection [73]. On the other hand, immunomodulatory medication (i.e., tocilizumab) and anticoagulant (i.e., heparin) have shown effectiveness in the prevention of death in critically ill patients [74–76]. These medications with strong evidence of efficacy have obtained approval or emergency use authorization from FDA [77]. Nonetheless, the current treatments for COVID-19 could be associated with adverse effects. For example, 23% of the COVID-19 patients treated with remdesivir suffered abnormality of hepatic functions, while 2% of the patients treated with tocilizumab developed septic shock [78, 79]. As the disease exacerbates, COVID-19 patients might suffer from hypoxemia or dyspnea. Hence, supportive treatment using high-flow oxygen is performed to maintain oxygen saturation in COVID-19 patients. In more severe cases, other respiratory therapies such as mechanical ventilation and endotracheal intubation are needed to alleviate severe respiratory symptoms [80].

COVID-19 patients who are immunocompromised or receiving immunosuppressive therapy are vulnerable to the infection as they are unlikely to mount adequate antibody responses through vaccination. Nonetheless, such patients can passively obtain immediate immunity by receiving anti-SARS-CoV-2 neutralizing antibodies from plasma donated by individuals who have recently recovered from the illness. A meta-analysis of clinical trials demonstrated that passive antibody therapy is associated with a reduction in mortality in immunocompromised COVID-19 patients [81]. Furthermore, a study showed that early administration of convalescent plasma to severe COVID-19 patients reduced the incidences of disease exacerbation and mortality [82].

In addition to modern treatments, complementary and alternative medicines have been integrated into the current management of COVID-19 in some countries [83]. In China, qingfei paidu decoction containing a mixture of 22 herbs has been used in the treatment of COVID-19, with an effective cure rate of over 90% [84]. While in India, ancient treatments such as Ayurvedic and Unani have been employed to combat the COVID-19 disease [85, 86].

The discovery of new antiviral therapies (i.e., paxlovid) and monoclonal antibodies (i.e., sotrovimab) designated for COVID-19 have shown evidence of benefit in reducing mortality and hospitalization rates [87, 88]. However, these studies were conducted before Omicron became dominant. A recent study by Aggarwal et al. [89] showed that the effectiveness of sotrovimab was attenuated in preventing the disease progression during Omicron BA.1 phase. On the other hand, paxlovid showed preliminary *in vitro* efficacy in treating the Omicron [90], but its efficacy in clinical trials remains to be elucidated. Also, these treatments are costly and with limited availability to meet the needs of the world. There is a need for cost-effective therapies that are easy to administer globally.

It is generally believed that the COVID-19 vaccine is needed to restore prepandemic normalcy. Currently, many COVID-19 vaccines have been developed using various vaccine platforms including live attenuated virus, recombinant viral-vectored vaccines, inactivated or killed virus, protein subunit vaccines, virus-like particles, and DNA or mRNA-based vaccines [91]. Among the developed vaccines, mRNA-based Pfizer-BioNTech's BNT162b2 and Moderna's mRNA-1273 vaccines have shown efficacy above 94% in clinical trials [92]. Mass COVID-19 vaccination has established herd immunity as evidenced by the drop in the morbidity and mortality rate following the vaccination [66, 114]. However, a study from Aye et al. [93] demonstrated that there is a temporal association between adverse cardiac manifestations and COVID-19 vaccines (i.e., Pfizer-BioNTech, Moderna, and Janssen), raising safety concerns and the probability of long-term side effects in the public interest.

## 6. Aptamers and Systematic Evolution of Ligands by Exponential Enrichment (SELEX)

**Figure 1:**
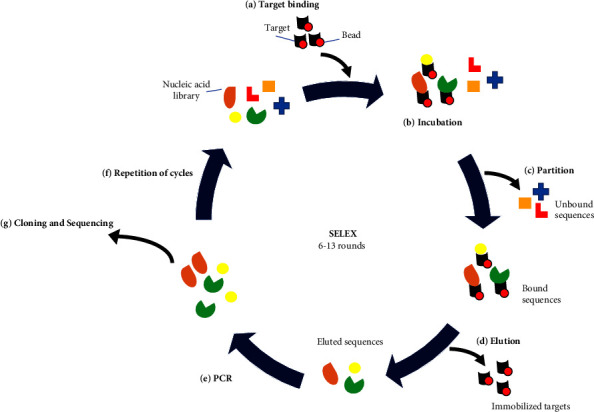
Selection of aptamers using the SELEX method: (a) the SELEX involves an iterative selection of ssDNA or RNA aptamers from a randomized oligonucleotide library where the selection starts by immobilizing the target of interest to beads, (b) the immobilized target of interest is then incubated with a randomized oligonucleotide library, (c) following incubation, the unbound oligonucleotides are separated from the bound aptamers, (d) the bound aptamers are then eluted from the immobilized targets and (e) amplified using PCR to create a new enriched selection pool, (f) the enriched pool serves as a starting library for the next SELEX cycle and the cycle is repeated for 6–13 rounds to achieve desired binding affinity, and (g) once the binding affinity is achieved, the aptamers of the last round are cloned and sequenced.

The aptamer is a short length of nucleic acids (RNA or single-stranded DNA (ssDNA)) which have a 3D conformation that is capable of binding to its target with high affinity and specificity. The aptamer is selected from a synthetic nucleic acid library through an *in vitro* selection called systematic evolution of ligands by exponential enrichment (SELEX) (Figure 1). The SELEX includes iterative cycles of binding of aptamers to a target, separation of bounded aptamers from unbound oligonucleotides, recovery of the bounded aptamers from the target, amplification of the bounded aptamers, and regeneration of RNA or ssDNA aptamers. Generally, after 6–13 rounds of the SELEX process, the initial unselected nucleic acid library is then dominated by a new population of aptamers that show higher affinity toward the target as the lower affinity aptamers are discarded in each round of SELEX. The final enriched aptamers are then cloned and sequenced for downstream analysis. The aptamers of interest can then undergo post-SELEX modifications such as truncation, chemical structural modification, or conjugation to other molecules to enhance their functional extremities [96].

## 7. Critical Factors Affecting Aptamers Discovery

Several key aspects of SELEX can influence the selection outcome to varying extents. To isolate aptamers with comparative binding affinity for future applications, consideration of the following aspects is necessary to ensure a successful SELEX process to identify target-specific aptamers with sufficient binding affinity.

## 8. Library Types

Selection of the initial types of the nucleic acid library is a fundamental step for SELEX. DNA- and RNA-based libraries are currently the most frequently used nucleic acid library. These libraries are composed of chemically modified and unmodified nucleotides [97]. The development of chemically modified nucleotides is to improve the functional characteristics and affinity binding of the aptamers. As aptamers are often applied in biological media full of nucleases (i.e., blood), unmodified nucleotides which are susceptible to nuclease degradation may reduce the half-life of the aptamers. Hence, several pre-SELEX nucleotide modification approaches are developed to increase the nuclease stability of the aptamers. These chemical modifications can be performed either on the sugar-phosphate backbone (i.e., changing phosphate to methyl phosphonate) or bases (i.e., 7-(2-thienyl) imidazo (4, 5-*β*) pyridine (Ds)) of the nucleotides [98]. Nonetheless, the isolation protocol of chemically modified libraries is much more complicated [99]. Hence, unmodified libraries are often used in the SELEX process and the functionality of the aptamers is improved in post-SELEX chemical modification approaches [96].

The function of both ssDNA and RNA aptamers is essentially the same, but each has its benefits. RNA aptamers can form more diverse functional motifs with stronger intrastrand RNA-RNA interactions [100]. However, several studies suggested that ssDNA exhibits a comparable propensity for forming intricate tertiary structures similar to RNA [101, 102]. ssDNA aptamers are also preferentially selected over their RNA counterpart as they are inherently more stable and the related manufacturing costs are lower [103, 104]. The majority of the reported aptamers specific to COVID-19 detection were also developed using DNA libraries (Tables 2 and 3).

## 9. Library Design

Generally, the starting library is made up of sequences of the same length with a random core that is flanked by a fixed primer binding region at each 5' and 3' end. The random core region forms the 3D structure which provides binding motif candidates to the target. This core region can be randomized with the integration of the four nucleotides (A, G, C, or T/U) at an equal proportion of 25% : 25% : 25% : 25% or at a predefined proportion biasing G or C bases. The uniform distribution of the four bases in the random core region provides maximal sequence diversity, thereby increasing the chances of isolating desired aptamers. Meanwhile, increasing the GC content to form pyrimidine-rich sequences can enhance the structural stability and complexity of the folded aptamer. For example, the computational analysis using an RNA library by Gevertz et al. [105] showed that increasing the GC bases to 30% each resulted in the formation of aptamers with more loops and stems.

A library with a random region of 25-nucleotide (either A, G, C, or T/U; 4^25^) comprising 10^15^ unique sequences is enough for successful aptamer isolation. Lozupone et al. [106] demonstrated that a 26-nucleotide randomized library can result in a faster selection process within 5 rounds compared to a 22-nucleotide randomized library that needed 7 rounds of selection. This finding suggests that a longer randomized library contains more diverse and complex sequences which can facilitate the selection speed (i.e., reduce the selection cycle). Moreover, a longer randomized region reduces the interference of the primer binding regions in the formation of 3D structures that can minimize the loss of desired aptamers during the polymerase chain reaction (PCR) amplification step [107]. Nonetheless, studies suggested the random region is optimized at 30–50 nucleotides as the synthesis of a longer oligonucleotide library is costly, time-consuming [108], and could lead to the formation of by-products during PCR [109]. The optimized random region length also ensures the efficiency of RNA regeneration as transcription from a longer DNA will be less effective [110, 111]. Practically, all the aptamers used in COVID-19 detection and treatment are in the range of 30–50 nucleotides (Tables 2 and 3).

## 10. Target Binding and Partitioning

SELEX is efficient when the aptamers with sufficient affinity are enriched within the minimal rounds of selection. Effective separation of the bound aptamers from the rest of the pool can ensure the enrichment of aptamers within the minimal round of selection as capturing the nonspecific sequences in each selection cycle can decelerate the enrichment process (Figure 1). Several strategies have been developed for bound/unbound sequence separation. These strategies can be divided into several groups based on their approaches: (i) coincubation of target and library in matrices-free condition (i.e., nitrocellulose membrane filtration and capillary electrophoresis), (ii) immobilizing targets on a solid phase (i.e., bead), and (iii) immobilizing sequences on a solid phase (i.e., nanoselection) [100]. Among these strategies, capillary electrophoresis and nanoselection allow enrichment of the desired aptamer within a single round of selection due to their high-separation resolution [112, 113]. Nonetheless, these methods need sophisticated apparatus. On the other hand, nitrocellulose membrane filtration is easy to handle and doable in regular laboratories, but this method is only suitable for protein [16]. Immobilizing targets on the bead is suitable for a broad range of targets, but nonspecific binding to the bead can occur. Furthermore, the nitrocellulose membrane filtration and bead partitioning methods have low separation efficiency, but can be overcome by carefully determining the target concentration and increasing the number of selection cycles to achieve a selection pool with adequate bulk dissociation constant [114]. Hence, the selection of the partitioning protocol should thus depend on the target's nature or size, and the availability of the related techniques and equipment in the lab.

In the search for aptamers for COVID-19 detection and treatment, the bead-based SELEX is the most popular protocol used (Tables 2 and 3). In these studies, the researchers performed a negative selection by incubating the selected pool with the bead to reduce the nonspecific binding in each round, and highly specific aptamers with high affinity were successfully isolated within 6 to 13 rounds of SELEX.

## 11. Amplification

The enrichment process of the desired aptamers happens through cyclic discarding of the unbound sequences and amplification of the bound aptamers over the course of SELEX selection (Figure 1). It is important to optimize the number of PCR cycles as PCR by-products are formed when there are nonspecific interactions between the primer-product, product-product, or primer-primer during the PCR amplification. Accumulation of the PCR by-products can lead to a greater tendency of PCR bias as the PCR preferentially amplifies shorter sequences [115]. Also, PCR amplification preferentially favors those sequences with lower structural stability (i.e., sequences with lesser GC bases) [116]. The use of emulsion PCR (ePCR) might be useful to eliminate the PCR bias by amplification of DNA molecules in picoliter-volume water-in-oil emulsion droplets. Villa et al. [10] implemented ePCR into their SELEX protocol to ensure an equal rate of amplification of each isolated aptamer against ACE2 protein [115]. Nevertheless, this method is expensive and cumbersome as it requires extensive amounts of DNA polymerase and repeated sequencing after each SELEX cycle [98].

## 12. Regeneration of ssDNA or RNA

After the PCR amplification, it is crucial to prepare the subsequent ssDNA/RNA selection pool from the double-stranded DNA (dsDNA) PCR product. Unlike the RNA which is mainly transcribed from the dsDNA using RNA polymerase [9], there are four common methods to regenerate the ssDNA from the parental dsDNA. These methods are (i) amplification of one strand of the parental template by the unequal molar ratio of forward and reverse primers through asymmetric PCR, (ii) separation of the forward and reverse sequence using denaturing urea polyacrylamide gel electrophoresis (Urea-PAGE), (iii) separation of desired sequences (unbiotinylated) from biotinylated sequences that are immobilized to the streptavidin-conjugated magnetic bead, and (iv) lambda exonuclease digestion of the 5′-phosphorylated strand of dsDNA [117].

In the search for aptamers targeting COVID-19, Schmitz et al. [7] used lambda digestion to create their ssDNA selection pool for the isolation of aptamer targeting the SARS-CoV-2 S protein, while Song et al. [4] used the streptavidin-coated beads to separate the desired sequence from the biotinylated sequence during the isolation of aptamer targeting the SARS-CoV-2 S_RBD_ protein. Innovatively, Villa et al. [10] used a 3-minute thermal denaturation at 94°C, followed by rapid cooling on ice for 5 min to prepare the ssDNA pool in the selection of aptamer against the ACE2 proteins. As the rapid cooling of the denatured dsDNA can prevent the rehybridization of the complementary strands, both the forward and reverse strands can be folded into secondary structures for aptamer selection. Nevertheless, there is a chance that the complementary strands are rehybridized and mute the possible aptamer [118].

## 13. Application of Aptamers in COVID-19 Detection

In seeking new detection methods for COVID-19, several studies have identified diagnostic aptamers that selectively target the structural protein of SARS-CoV-2 (Table 2). Most of the studies have mainly selected the N protein as the target as the N protein is the most abundant structural protein in the early stage of the infection [119, 120] and can be detected in various human samples such as nasopharyngeal aspirate, urine, and fecal [2].

To search for aptamers with high specificity for COVID-19 detection, Zhang et al. [5] coincubated their enriched ssDNA pool with Salmon extract double-stranded DNA and N protein to eliminate those nonspecific and low-affinity ssDNA aptamers [5]. With this, they have successfully obtained four SARS-CoV-2 nucleocapsid protein-specific ssDNA aptamers, all with a dissociation constant (*K*_D_) in the low range of nanomolar.

Zhang et al's [5] work also proved that the aptamer is a rapid and robust diagnostic tool where the identified nucleocapsid protein-specific aptamers can detect the target within 15 minutes across different types of human samples such as urine and serum with a detection limit of 1 ng/mL. The rapid detection is large because aptamers are small and induce a less steric hindrance on their target surface that could govern the reactivity of the aptamers to bind to their target readily [121]. Moreover, because they are small, aptamers only occupy a small space on the surface of the target. This phenomenon allows the target to provide more recognition domains for the isolation of several aptamers that target different domains [5]. The high programmability of the aptamers then allows the construction of multivalent aptamers which are shown to be more sensitive and reactive than the monovalent aptamer [9, 96].

Some aptamers have been incorporated into aptasensing platforms to offer cost-effective alternatives to existing molecular diagnostics tools for COVID-19 [122, 123]. The biotin-streptavidin methodology is one of the methods used for the attachment of aptamer in optical aptasensors. In brief, biotin is first immobilized onto a solid surface followed by streptavidin coating. The streptavidin layer is then used to immobilize the biotinylated aptamer in the detection tool [122]. The aptamer against the spike protein RBD isolated by Song et al. [4] has been used to develop a surface plasmon resonance-based, D-shaped plastic optical fiber aptasensor and a surface-enhanced Raman scattering-based detection assay [124, 125]. Moreover, a highly sensitive and specific aptamer-linked immobilized sorbent assay (ALISA) has been developed by an Indian company THSTI for SARS-CoV-2 detection [123]. A portable aptamer-based nanosensor created by the Berkeley-based Pinpoint Science laboratories demonstrates the potential to replace or supplement existing detection tools by its ability to produce results within 30 seconds. However, further trials with large sample size are required to assess the reliability of these aptasensors [123].

## 14. Application of Aptamers in COVID-19 Treatment

The ability of the aptamer to fold into a 3D structure that can fit its target with high affinity suggests its potential in therapeutical application. Moreover, several significant characteristics of aptamers had put them on the verge of becoming a new class of pharmacological agents. The aptamer (∼2 nm) is smaller in size than the antibody (∼10 nm) which allows it to target difficult-to-reach binding sites. Also, the aptamer elicits a very low immune response, and its high programmability allows post-SELEX modification on its structure to improve its stability under different physiological conditions. Considering these distinct advantages, aptamers have been validated as potential remedies for several diseases [126]. For example, FDA approved the first RNA aptamer, pegaptanib to treat ocular vascular disease by targeting vascular endothelial growth factor (VEGF) [127].

In search of novel drug candidates, several research groups have targeted aptamer blocking strategy as a new direction to fight against the current COVID-19 infection. The key elements in the viral entry pathway of the SARS-CoV-2 are therefore the interesting targets of aptamers, including the viral S protein and host ACE2 protein, as shown in Table 3. Except for the aptamer reported by Parashar et al. [3] which is a repurposing aptamer that was intended against SARS-CoV, all the aptamers listed in Table 3 were identified specifically for SARS-CoV-2. Most of the research groups independently targeted the RBD of S protein in their aptamer isolation as the RBD is the contact point of virus-host cell interaction [21]. On the other hand, isolated aptamer specifically bound to ACE2 which is recognized by the RBD may display broad neutralizing potential against other coronaviruses [10]. Schmitz et al. [7] identified an ssDNA aptamer that specifically interacts with the S protein, not the RBD. Although this aptamer did not block the interaction between the S protein and ACE2, its binding to other domains of the S protein showed potent inhibition in pseudoviral infection, suggesting an unknown infection modality of the SARS-CoV-2 [7].

These aptamers displayed significant inhibitory properties in the tissue culture where their inhibitory efficacy is largely attributed to the low dissociation constant of the isolated aptamers that are in a low range of nanomolar (Table 3). With the reported binding affinity of the spike proteins of different SARS-CoV-2 variants toward the ACE2 which falls in the range of 5–30 nM [128], the comparable binding affinity of these isolated aptamers may interfere in the virus-receptor binding interaction through several mechanisms such as prevention, substitution, or competition [8]. Despite the reality that the inhibitory properties of the aptamers have not been evaluated in animal models, their therapeutic application can be foreseen especially after post-SELEX modification on the structure of the aptamers that could enhance their functional extremities in physiological conditions [96].

## 15. Conclusion

**Figure 2:**
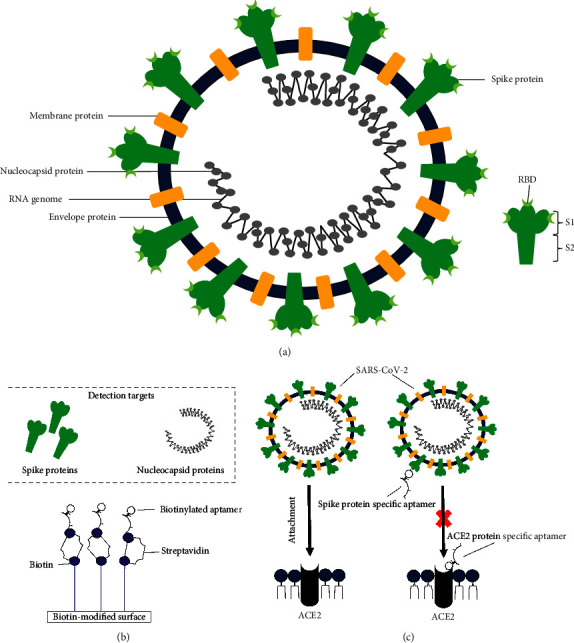
SARS-CoV-2 structure and the targets of aptamers in the detection and therapeutic of COVID-19. (a) Schematic diagram of the SARS-CoV-2 structure: The membrane, envelope, and spike proteins of SARS-CoV-2 are embedded in the viral envelope, which contains the viral RNA coiled with the N proteins. The trimeric spike protein can be divided into S1 and S2. The S1 consists of the RBD responsible for host receptor binding. (b) Schematic diagram of aptamer-based detection: spike proteins and nucleocapsid proteins are the main targets for COVID-19 detection. The aptamers recognizing these proteins have been biotinylated to attach to a biotin-modified surface using streptavidin to develop optical aptasensors for COVID-19 diagnostics. (c) Schematic diagram of aptamer blocking therapeutic strategy: the key elements involved in the viral entry pathway such as spike protein and host ACE2 protein are the interesting targets of therapeutic aptamers for COVID-19.

The uniqueness of aptamers has made them promising novel diagnostic and therapeutic agents in this COVID-19 pandemic. The aptamers (with the randomized region of 30–50 nucleotides) for SARS-CoV-2 were successfully selected using bead-based SELEX against the viral and/or host proteins (Figure 2). The SARS-CoV-2 N protein-specific aptamer has been incorporated with biosensor technologies to increase detection sensitivity. Also, the aptamer targeting the SARS-CoV-2 S protein has the potential to become a direct-acting antiviral and the aptamer against the host ACE2 protein could be a broad-spectrum inhibitor against any existing or future emerging viruses that also use ACE2 for cell entry in the future. The aptamer characteristics have shed commercial value in overcoming the antibody limitations in the development of diagnostics and therapeutics to address the current or future emerging infection crisis.

## Figures and Tables

**Table 1:** Pathophysiology at different disease phases of COVID-19 infection and their respective treatments.

Disease phase	Asymptomatic	Mild	Moderate	Severe	Critical	References
Disease mechanisms	Viral replication			Inflammation and hypercoagulability		Bae et al. [94]

Clinical manifestation	Positive for SARS-CoV-2 test but show no symptoms	Show symptoms of acute upper respiratory tract infection (i.e., fever, cough, and fatigue) and digestive symptoms (nausea, vomiting, stomachache, and diarrhea)	Show clinical or radiographic evidence of lower respiratory disease with no hypoxemia (i.e., oxygen saturation above 92%)	Show clinical or radiographic evidence of lower respiratory disease and hypoxemia (i.e., oxygen saturation below 92%)	ARDS, septic shock, and/or multiorgan dysfunction	Yuki et al. [95]

Possible treatments	(i) Antiviral			(i) Immunomodulator		Whittle et al. [80, 83, 94]
	(ii) Passive antibody therapy			(ii) Anticoagulation		
	(iii) Complementary and alternative medicine			(iii) Respiratory therapy		

FDA approved drugs	(i) Veklury (remdesivir)–—adult and pediatric patients			(i) Actemra (tocilizumab)–—adult patients		FDA [77]
				(ii) Olumiant (baricitinib)–—adult patients	

FDA emergency use authorization	(i) Paxlovid (nirmatrelvir and ritonavir)–—adult patients			(i) Kineret (anakinra)–—adult patients		FDA [77]
	(ii) Lagevrio (molnupiravir)–—adult patients			(ii) Olumiant (baricitinib)–—pediatric patients		
				(iii) Actemra (tocilizumab)–—pediatric patients		
				(iv) Gohibic (vilobelimab)–—adult patients		

**Table 2:** List of high-affinity ssDNA and RNA aptamers targeting N protein or RBD of the S protein isolated using different SELEX protocols as diagnostic agents for COVID-19.

Target	Types	Aptamer sequence (5′-3′)	Randomization region length (bp)	*K*_*D*_ (nM)	Partitioning method	Selection pool regeneration method	References
N Protein	ssDNA	GCAATGGTACGGTACTTCCGGATGCGGAAACTGGCTAATTGGTGAGGCTGGGGCGGTCGTGCAGCAAAAGTGCACGCTACTTTGCTAA	45	—	—	—	Chen et al. [2]

N Protein	ssDNA	GCTGGATGTCGCTTACGACAATATTCCTTAGGGGCACCGCTACATTGACACATCCAGC	36	0.49 ± 0.05	—	—	Zhang et al. [5]
		GCTGGATGTCACCGGATTGTCGGACATCGGATTGTCTGAGTCATATGACACATCCAGC		0.70 ± 0.06			
		GCTGGATGTTGACCTTTACAGATCGGATTCTGTGGGGCGTTAAACTGACACATCCAGC		2.74 ± 0.08			
		GCTGGATGTTCATGCTGGCAAAATTCCTTAGGGGCACCGTTACTTTGACACATCCAGC		4.38 ± 0.06			

S_RBD_	ssDNA	CAGCACCGACCTTGTGCTTTGGGAGTGCTGGTCCAAGGGCGTTAATGGACA	40	5.8	Bead	Magnetic separation using streptavidin-coated bead	Song et al. [4]
		ATCCAGAGTGACGCAGCATTTCATCGGGTCCAAAAGGGGCTGCTCGGGATTGCGGATATGGACACGT		19.9			

**Table 3:** List of high-affinity ssDNA and RNA aptamers targeting the S protein, RBD of the S protein, N protein, or ACE2 protein isolated using different SELEX protocols as potential therapeutic agents for COVID-19.

Target	Types	Aptamer sequence (5′-3′)	Randomization region length (bp)	*K*_*D*_ (nM)	Partitioning method	Selection pool regeneration method	References
S Protein	ssDNA	GGGAGAGGAGGGAGATAGATATCAACCCATGGTAGGTATTGCTTGGTAGGGATAGTGGGCTTGATGTTTCGTGGATGCCACAGGAC	40	21 ± 4.6	Bead	Lambda exonuclease digestion	Schmitz et al. [7]

S Protein	ssDNA	ATCCAGAGTGACGCAGCATCGAGTGGCTTGTTTGTAATGTAGGGTTCCGGTCGTGGGTTGGACACGGTGGCTTAGT	40	28	Bead	—	Liu et al. [6]

S_RBD_	ssDNA	CGCAGCACCCAAGAACAAGGACTGCTTAGGATTGCGATAGGTTCGG	—	44.78 ± 9.97	Bead	—	Sun et al. [8]

S_RBD_	RNA	GATCCATGGGCACTATTTATATCAACCTCTTCCTGACGGAAGTATCGGTCCAGGAATTACAAATGTCG TTGGTGGCCC	36	∼18	Bead	RNA polymerase transcription	Valero et al. [9]
		ATCCAGAGTGACGCAGCAATTTACCGATGGCTTGTTTGTAATGTAGGGTTCCGTCGGATTGGACACGGTGGCTTAGT		27			

N protein	RNA	UGUCGUUCGCUGUCUUGCUACGUUACGUUACACGGUUGG	—	1.65 ± 0.41	—	—	Parashar et al. [3]

ACE2	ssDNA	ATAGTCCCTGGCGTGCTTGACAGCAACACGAACACTGAACGTTCTTAACAAGCCTGGCAGAGCAGGTACGGTGTCA	40	33	Bead	Denaturation and rapid cooling	Villa et al. [10]
		ATAGTCCCTGGCGTGCTTCCGCGAGAGTTCGACATTCCCGAAAGTACGACGAAAGTCCAGAGCAGGTACGGTGTCA		47			

## Data Availability

The data supporting this review are from previously reported studies and datasets, which have been cited.
